# State-Level Economic Costs of Fatal Injuries — United States, 2019

**DOI:** 10.15585/mmwr.mm7048a2

**Published:** 2021-12-03

**Authors:** Cora Peterson, Feijun Luo, Curtis Florence

**Affiliations:** 1 Division of Injury Prevention, National Center for Injury Prevention and Control, CDC.

Unintentional and violence-related injury fatalities, including suicide, homicide, overdoses, motor vehicle crashes, and falls, were among the 10 leading causes of death for all age groups in the United States in 2019.* There were 246,041 injury deaths in 2019 (unintentional injury was the most frequent cause of death after heart disease and cancer) with an economic cost of $2.2 trillion (*1*). Extending a national analysis (*1*), CDC examined state-level economic costs of fatal injuries based on medical care costs and the value of statistical life assigned to 2019 injury records from the CDC’s Web-based Injury Statistics Query and Reporting System (WISQARS).^†^ West Virginia had the highest per capita cost ($11,274) from fatal injury, more than twice that of New York, the state with the lowest cost ($4,538). The five areas with the highest per capita total fatal injury costs were West Virginia, New Mexico, Alaska, District of Columbia (DC), and Louisiana; costs were lowest in New York, California, Minnesota, Nebraska, and Texas. All U.S. states face substantial avoidable costs from injury deaths. Individual persons, families, organizations, communities, and policymakers can use targeted proven strategies to prevent injuries and violence. Resources for best practices for preventing injuries and violence are available online from the CDC’s National Center for Injury Prevention and Control.^§^

The economic cost estimate for injuries that occurred in 2019 uses the societal perspective, including tangible and intangible costs to multiple payers. Costs are presented in 2019 U.S. dollars (USD). WISQARS fatal injury counts are from CDC’s National Vital Statistics System mortality data. In 2019, approximately 70% of U.S. injury deaths (173,040) were attributable to unintentional injuries (among which 36% were related to drug poisoning, 23% to falls, and 22% to motor vehicle traffic); approximately 20% were suicides; and 8% were homicides.^¶^ Medical costs were adjusted for patient characteristics (*2*), including comorbidities, sex, and age, and were modified to 2019 USD** and assigned to WISQARS records by injury mechanism (e.g., fall), intent (e.g., unintentional), and place of death (e.g., inpatient hospital). Aggregated medical costs (e.g., combined intents by mechanism or combined mechanisms by place of death) from reference sources were assigned when specific estimates by intent or mechanism were not available. The average medical cost among 2019 injury deaths was approximately $15,400^††^; however, many injury deaths had lower costs because the deaths occurred outside a health care setting (*2*).

The cost of injury mortality includes value of statistical life, a monetary estimate of the collective value that persons place on mortality risk reduction as derived in research studies through revealed preferences (e.g., observed wage differences for dangerous occupations) or stated preferences from surveys of persons’ willingness to pay for mortality risk reduction (*3*). Value of statistical life estimates were assigned by decedent age: 0–17 years, $16.9 million (*4*); 18–65 years, $10.7 million (*3*); values descending from $6 million (aged 66 years) to $410,000 (aged ≥100 years), reflecting the estimate for persons aged 18–65 years adjusted for older adults’ decreasing general life expectancy and baseline quality of life. Per capita fatal injury costs by U.S. state are presented graphically by injury intent (Figure). Injury fatality rates by state, intent, sex, and age group (0–24 years, 25–64 years, and ≥65 years) were examined to better understand the contributing circumstances in states with the highest per capita total injury costs. All reported data can be queried online using WISQARS. This activity was reviewed by CDC and was conducted consistent with applicable federal law and CDC policy.^§§^

**FIGURE:**
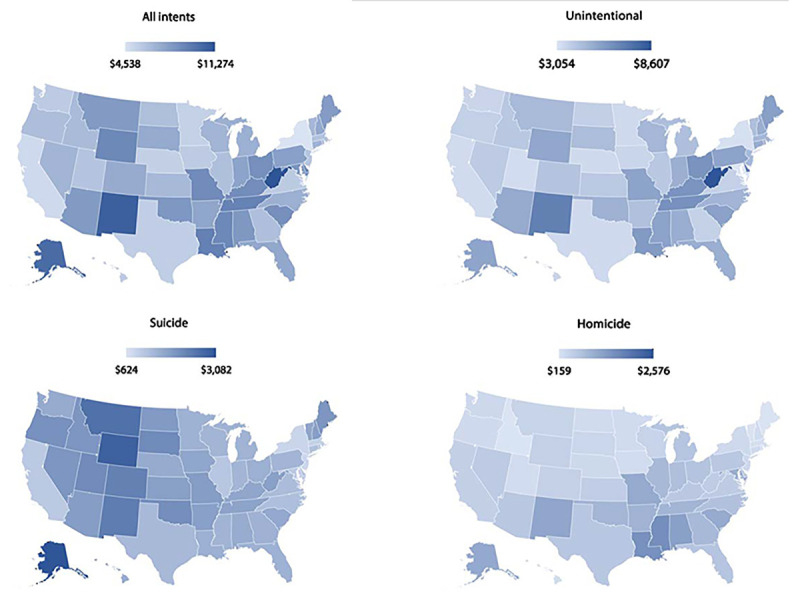
State-level fatal injury costs*^,^^†^ per capita, by intent — United States, 2019 **Abbreviation:** USD = U.S. dollars. * 2019 USD. ^†^ https://wisqars.cdc.gov/cost

The five areas with the highest per capita total fatal injury costs were West Virginia, New Mexico, Alaska, DC, and Louisiana; those with the lowest costs were New York, California, Minnesota, Nebraska, and Texas (Figure).^¶¶^ West Virginia had the highest per capita cost ($11,274) from fatal injury, more than twice that of New York, the state with the lowest cost ($4,538). The five states with the highest per capita costs for unintentional injury deaths were West Virginia, New Mexico, Delaware, Tennessee, and Ohio; the states with the lowest costs were Maryland, New York, Utah, California, and Nebraska. Per capita unintentional injury cost in West Virginia ($8,607) was approximately triple that in Maryland ($3,054). The five states with the highest per capita costs for suicide were Alaska, Wyoming, Montana, New Mexico, and Colorado; those areas with the lowest costs were DC, New Jersey, New York, Massachusetts, and Maryland. Per capita suicide cost in Alaska ($3,082) was approximately five times than that in DC ($624). The five areas with the highest per capita costs for homicide were DC, Mississippi, Louisiana, Alabama, and New Mexico; the states with the lowest costs were Idaho, Maine, Vermont, Rhode Island, and Massachusetts. Per capita homicide cost in DC ($2,576) was more than 16 times as high as that in Idaho ($159).

State-level injury fatality rates in 2019 by intent, sex, and age group suggest that the five areas with the highest per capita total fatal injury cost face different challenges in injury prevention. West Virginia had the highest age-adjusted unintentional injury fatality rate in the nation (including the highest for males, females, and persons aged 25–64 years) and the second highest for persons aged ≥65 years.*** New Mexico had the second highest age-adjusted unintentional injury fatality rate (including the second highest for males and females and third highest for persons aged 25–64 years) and was among the five states with the highest age-adjusted rate of both suicide (including for males, females, and each of the three assessed age groups) and homicide deaths (including for females and persons aged 25–64 years). Alaska had the nation’s second highest age-adjusted suicide rate (including for males, females, and persons aged 25–64 years, and the highest rate for persons aged 0–24 years) and the highest age-adjusted homicide rate for females. DC had the lowest age-adjusted suicide rate among all areas but the highest homicide rate (including for males and persons aged 0–24 and 25–64 years). Louisiana was among the top three states for the highest rate of age-adjusted homicide deaths (including for males, females, and each of the three assessed age groups).

## Discussion

All U.S. states face substantial avoidable costs from injury deaths. Identifying the economic cost of injuries is an essential part of the public health approach to injury and violence prevention and can support identification of cost-effective interventions. These findings highlight the need for targeted prevention strategies to achieve long-term value, or even cost savings, by preventing injury morbidity and mortality through addressing the causes of unintentional and violence-related injuries at the individual person, family, organizational, and community levels.

The number of injury deaths and a higher proportion of younger decedents had the biggest impact on each state’s total fatal injury cost. The cost of medical care for 2019 injury fatalities is marginal in comparison to the value of statistical life (*1*), which aims to capture complex costs related to mortality. Value of statistical life represents a value that is approximately 10 times higher than the value attributed to mortality based on foregone employment compensation, which was used to estimate states’ economic cost of fatal injuries in 2014 (*5*). At that time, similar to the results presented in this report, Alaska, Louisiana, New Mexico, Oklahoma, and West Virginia had the highest per capita total injury costs among U.S. states.

The findings in this report are subject to at least four limitations. First, although injury-related medical care incurs costs to specific, identifiable payers (including individual persons, health insurance payors, and employers), value of statistical life aims to capture costs of mortality that are not readily identifiable through financial transactions and thus are not as visible to some stakeholders. Second, available average medical costs in reference sources were not state-specific. Third, on the basis of available data, this study assigned value of statistical life by age group; however, the relationship between value of statistical life and age (and in particular, value of statistical life for older adults) is likely more complex than applied here and would benefit from further direct study (*6*). Finally, this report provides an initial assessment of states’ economic costs of injury by intent. Observed differences in per capita total fatal injury costs likely reflect important differences in affected populations (e.g., children, youths, and young adults versus older adults) and injury mechanism (e.g., firearm, fall, or drug poisoning) that must be understood to effectively target prevention resources.

Individual persons, families, organizations, communities, and policymakers can use targeted proven strategies to prevent injuries and violence. Data and resources that can assist in measuring and preventing injuries and violence, including suicide, overdoses, falls, firearm violence, motor vehicle crashes, traumatic brain injuries, adverse childhood experiences, youth violence, sexual violence, and intimate partner violence, are available online from CDC’s National Center for Injury Prevention and Control. Opportunities to investigate national and state-level injury data and costs are available online from WISQARS.

**Summary**

**What is already known about this topic?**

In 2019, injuries accounted for 246,041 U.S. deaths; the economic cost of these injuries was $2.2 trillion.

**What is added by this report?**

West Virginia had the highest per capita cost ($11,274) from fatal injury, more than twice that of New York, the state with the lowest cost ($4,538). The highest per capita fatal injury costs occurred in Alaska, District of Columbia, Louisiana, New Mexico, and West Virginia; the lowest occurred in California, Minnesota, Nebraska, New York, and Texas.

**What are the implications for public health practice?**

All states face substantial avoidable costs due to injury deaths. Resources for best practices for preventing injuries and violence are available online from CDC’s National Center for Injury Prevention and Control.

## Footnotes

*
https://wisqars.cdc.gov/data/lcd

†
https://www.cdc.gov/injury/wisqars

§
https://www.cdc.gov/injury

¶ Data available at https://wisqars.cdc.gov/data/explore-data/home. To access injury-related deaths by mechanism or intent for 2019, select 2019 as both the From and To years and then click the Explore Data Button. When the visualization appears, click the Filter Data button to view data by injury mechanism or intent.

** U.S. Bureau of Economic Analysis. National Income and Product Accounts: Table 2.5.4: Price Indexes for Personal Consumption Expenditures by Function (37. Health); 2020. https://www.bea.gov/itable (Accessed August 3, 2020).

†† Data available at https://wisqars.cdc.gov/cost and can be accessed using the following data selections: Injury Outcome = Fatal: Data Year = 2019; Mechanism = All Injury; Intent = All Intents; Geography = United States; Age Groups = 0 to 4 to Unknown; Sex = Males, Females, and Unknown; Cost Measure = Average; Cost Type = Medical Costs; and Report Layout Down/Row = None.

§§ 45 C.F.R. part 46, 21 C.F.R. part 56; 42 U.S.C. Sect. 241(d); 5 U.S.C. Sect. 552a; 44 U.S.C. Sect. 3501 et seq.

¶¶ Data available at https://wisqars.cdc.gov/cost and can be accessed using the following data selections: Injury Outcome = Fatal; Data Year = 2019; Mechanism = All Injury; Intent = All Intents; Geography = United States; Age Groups = 0 to 4 to Unknown; Sex = Males, Females, and Unknown; Cost Measure = Per Capita; Cost Type = Combined Costs; and Report Layout Down/Row = State and Intent (or State only for All Intents).

## References

[1] Peterson C, Miller GF, Barnett SBL, Florence C. Economic cost of injury—United States, 2019. MMWR Morb Mortal Wkly Rep 2021;70:1655–9. 10.1136/injuryprev-2019-043544 34855726 PMC8641568

[2] Peterson C, Xu L, Florence C. Average medical cost of fatal and non-fatal injuries by type in the USA. Inj Prev 2021;27:24–33. 10.1136/injuryprev-2019-043544 31888976 PMC7326639

[3] Office of the Assistant Secretary for Planning and Evaluation. Guidelines for regulatory impact analysis. Washington, DC: US Department of Health and Human Services, Office of the Assistant Secretary for Planning and Evaluation; 2016. https://aspe.hhs.gov/sites/default/files/migrated_legacy_files//171981/HHS_RIAGuidance.pdf

[4] Office of Child Care, Administration for Children and Families, US Department of Health and Human Services. Child Care and Development Fund (CCDF) program. Final rule. Fed Regist 2016 Sep30;81(190):67438–595. 27726322

[5] Luo F, Florence C. State-level lifetime medical and work-loss costs of fatal injuries—United States, 2014. MMWR Morb Mortal Wkly Rep 2017;66:1–11. 10.15585/mmwr.mm6601a1 28081055 PMC5687268

[6] Aldy JE, Viscusi WK. Adjusting the value of a statistical life for age and cohort effects. Rev Econ Stat 2008;90:573–81. 10.1162/rest.90.3.573

